# Interventions at the end of life – a taxonomy for ‘overlapping consensus’

**DOI:** 10.12688/wellcomeopenres.10722.1

**Published:** 2017-02-02

**Authors:** David Clark, Hamilton Inbadas, Ben Colburn, Catriona Forrest, Naomi Richards, Sandy Whitelaw, Shahaduz Zaman

**Affiliations:** 1 School of Interdisciplinary Studies, University of Glasgow, Glasgow, UK; 2 School of Humanities, University of Glasgow, Glasgow, UK

**Keywords:** end of life, interventions, global death, overlapping consensus

## Abstract

*Context:* Around the world there is increasing interest in end of life issues. An unprecedented number of people dying in future decades will put new strains on families, communities, services and governments.  It will also have implications for representations of death and dying within society and for the overall orientation of health and social care. What interventions are emerging in the face of these challenges?

*Methods:* We conceptualize a comprehensive taxonomy of interventions, defined as ‘organized responses to end of life issues’.

*Findings:* We classify the range of end of life interventions into 10 substantive categories: policy, advocacy, educational, ethico-legal, service, clinical, research, cultural, intangible, self-determined. We distinguish between two empirical aspects of any end of life intervention: the ‘locus’ refers to the space or spaces in which it is situated; the ‘focus’ captures its distinct character and purpose. We also contend that end of life interventions can be seen conceptually in two ways – as ‘frames’ (organized responses that primarily *construct* a shared understanding of an end of life issue) or as ‘instruments’ (organized responses that *assume* a shared understanding and then move to act in that context).

*Conclusions:* Our taxonomy opens up the debate about end of life interventions in new ways to provide protagonists, activists, policy makers, clinicians, researchers and educators with a comprehensive framework in which to place their endeavours and more effectively to assess their efficacy. Following the inspiration of political philosopher John Rawls, we seek to foster an ‘overlapping consensus’ on how interventions at the end of life can be construed, understood and assessed.

## Context

The world is facing an unprecedented level of dying, death and bereavement, even when compared to the periods of global war in the last century. Indeed, more people are living today in the world than died in the entire twentieth century. Globally, around 56 million people die each year, a figure that could perhaps reach 90 million by mid-century ^1^. What does this mean for global health discourse, public debate, planning and the delivery of appropriate end of life services? How well advanced are individual states in their thinking, disposition and preparedness for human death at these levels? How will attitudes, social practices, norms and expectations relating to dying and death be shaped and transformed? In particular, what are the prospects for sustainable, appropriate and effective forms of intervention at the end of life?

End of life issues should constitute an important aspect of global health discourse ^2^. As shown by the World Health Organization (WHO), low-income countries account for about 80% of deaths from non-communicable disease, 99% of annual maternal deaths, around 95% of tuberculosis deaths, children from the poorest 20% of households are nearly twice as likely to die before their fifth birthday as children in the richest 20%, and life expectancy can vary by as much as 36 years between the richest and poorest countries (WHO. *Ten Facts on Health Inequities and their Causes*. 2011: www.who.int/features/factfiles/health_inequities/en/; accessed 25 January 2017). The Global Forum for Health Research refers to the “10/90 gap” ^3^: less than 10% of worldwide resources are devoted to health research in developing countries, yet these countries are where over 90% of all preventable deaths occur worldwide. Although the term originally emphasized the research disparity, it is now widely used to convey wider global health differences ^4^.

This gap is clearly evident in the scenario of end of life care. The highest proportion (78%) of adults who could benefit from palliative care at the end of life are in low and middle-income countries, but the most developed levels of palliative care provision are found in the higher-income countries, as shown in the WHO and Worldwide Palliative Care Alliance *Global Atlas of Palliative Care at the End of Life*. (London: Worldwide Palliative Care Alliance; 2014: www.who.int/nmh/Global_Atlas_of_Palliative_Care.pdf; accessed 25 January 2017). The vast majority (98%) of children in need of palliative care at the end of life belong to low and middle-income countries ^5^. Inequitable access to pain control is a specific example. High-income countries account for nearly 92% of medical morphine consumed in the world, but comprise only 17% of the total population. In contrast, low and middle-income countries, representing the remaining 83% of the world’s population, account for a mere 8% of total medical morphine consumption ^6^. Despite the number of people dying in low and middle income settings, very little is known from a research perspective about how palliative and end of life care are being structured and delivered there. Systematic reviews show that 90% of palliative care studies focus on just a few specific European countries ^7^, and that most international palliative care research is taking place in high income contexts ^8^. Likewise, global mapping of the levels of palliative care development shows wide variation in the preparedness and capacity of health systems for palliative care delivery in developed and developing countries, with only around 20 countries demonstrating a high level of integration of end of life provision with the wider systems of health and social care ^9^.

In addition to acknowledging death as something that must be faced, there is also an ongoing need to avoid early mortality when this can be done. There are negative associations for ‘palliative care’ when it is seen as a substitute for effective prevention and cure of disease ^10^. We detect a growing discourse on end of life issues, wherein ‘how we die’ has become a contested space of pluralistic viewpoints, oppositional arguments and sometimes confusing terminologies and categories. This is compounded by changing epidemiological patterns of illness, ageing and disability.

An increasing proportion of those who die will not do so suddenly. For them death will come at the end of a long life and might also be accompanied by a ‘long goodbye’. Chronic illness and frailty have become common hallmarks of the last years of life in the context of population ageing. The phenomenon of ageing is increasingly prevalent even in countries that currently have lower life expectancies; global population growth is in turn being driven by ageing ^11^. In this context – of high demand for services and resources, and complex, protracted clinical need - it is unclear if the end of life can be widely experienced with dignity, compassion, fairness and freedom from pain and distress. Whilst end of life issues are beginning to figure more strongly in public debate and planning, we still have limited examples of innovation and imagination that can inspire new approaches to these matters, beyond those that sit within the paradigm of hospice, palliative care and related health and social services. Recently, the advocates of compassionate cities ^12^ and compassionate communities ^13, 14^ place an alternate emphasis on the power of citizens to combine their efforts to generate cultures of care that complement, or may even compensate for, the inadequacies of formal systems. These may have some traction in neo-liberal policy contexts ^15^, but so far are largely exhortatory and still under-researched.

At the same time it is argued, for example by the Worldwide Hospice Palliative Care Alliance, that the quest for a global health system offering universal health coverage should include palliative care as a fundamental goal ( *Universal Health Coverage and Palliative Care: Do not leave those suffering behind*. London: Worldwide Hospice Palliative Care Alliance; 2014: www.thewhpca.org/resources/item/uhc-and-palliativecare; accessed 25 January 2017).

Similarly, the World Health Assembly has called on all its member states to acknowledge palliative care within health policies and to make provision at the community level and across the life course ^16^. Consequently, there is much talk of the ‘gap’ between those who need pain relief and those who access it, and between those who can benefit from palliative care and those who can obtain it. More diffuse and difficult to determine are the gaps that exist between cultures, countries and constituencies of many kinds in their orientation to end of life issues, and the most appropriate responses that should result ^17^.

One important way to bridge these gaps is through mutual learning between people, practices and approaches around the world. Increasingly the philosophies and attitudes relating to such partnerships seek a two-way flow of expertise and knowledge ^18^. Such innovation can be sourced globally, and there are opportunities for the co-development of ideas that can spread from richer to poorer countries and vice versa. ‘Reverse innovation’, defined as the flow of ideas from low to high income settings, has gained momentum in the business world, and recently has been applied to the healthcare setting ^19^. The processes for supporting ‘reverse’ or ‘frugal’ innovation can be in the form of partnerships between institutions in the global north and south and, increasingly in south to south partnerships. A strong commitment to valuing different forms of knowledge is required to promote learning that challenges and rethinks entrenched practice within global systems ^20^. Blending global knowledge with on-the-ground innovations, particularly from developing countries, may serve to transform future modes of international cooperation. More needs to be done to support this reverse flow, for two reasons. First, there is a danger of missing out on the ideas of more than half the world. Second, and perhaps even more importantly, there is the risk of ignoring the extraordinary potential of learning from people and countries that frequently do not have established or bureaucratic infrastructures for health and healthcare, and therefore may have greater freedom to experiment and innovate.

In this context of marked inequalities in health, mortality and end of life care worldwide, and the growing interest in mutual learning and ‘reverse innovation’, we set out here a taxonomy of end of life interventions. We seek to develop a more structured and in-depth understanding of different kinds of interventions around the world, which will complement a more organized mapping of inequities in this field. The taxonomy provides a conceptual means for stakeholders in any setting to chart their existing interventions, which might in turn facilitate ‘reverse innovation’, mutual sharing and responsible innovation ^21^.

In doing this, we take an inclusive view of interventions at the end of life. We define them broadly as ‘organized responses to end of life issues’. This requires us to see the level of ‘organization’ in various ways. It can be formally constituted in bureaucratic structures and processes that are documented, defined and regulated. End of life services, legal procedures and policies cluster around this ‘pole’ of organization. At the same time ‘organized responses’ are visible in the absence of formal regulatory mechanisms. They can be found in the space between persons or in the informal responses of relatives to the needs of a family member. They can cluster around social actions, events, and expressions or phenomena that give artistic form to end of life issues.

In short, there is potentially a rich array of end of life interventions that merit attention. We set out here some propositions for classifying them and explore the potential benefits that might then result.

## Objectives

The main objective of this work is to generate a taxonomy of end of life interventions wherein they are ordered and named in a system of classification. This in turn has four distinct benefits: 1) it provides a platform from which to gain an overview of the many forms of organized human endeavour that are oriented towards end of life issues; 2) it offers a way to map this terrain into relatively distinct elements; 3) it alerts sectional interests and specific stakeholders involved in end of life interventions to the range of cognate actions that exist, thus providing opportunities for synergy, partnership or complementarity; 4) it provides a framework for the macro-evaluation and synthesis of knowledge across the spectrum of extant interventions.

We aim to conceptualize a taxonomy that is useful both for practice and for theoretical analysis.

## Methods

Our approach has been to reflect on the diversity of organized responses to end of life issues that exist in different contexts. The social science tradition has tended to distinguish between ‘taxonomy’ (as empirical) and ‘typology’ (as conceptual) ^22^. This is not a distinction we are inclined to accept. Instead we suggest that classification, particularly of emergent and disputed phenomena (which are features of many end of life interventions), proceeds more effectively by bringing the empirical together with the conceptual, in order to forge a particular understanding and codification. We refer to this as ‘taxonomy’, but it does not exclude conceptualisation.

We have constructed our taxonomy as a way of responding to and illuminating the diverse activity our exploration has uncovered.

It is worth elaborating a little on our methodology here. The paradigm taxonomies are biological and botanical theories of species, in which fauna and flora are named and described, and on the basis of various different characteristics are then given a place in a hierarchical structure of classifications. More generally, when we construct taxonomy we create a conceptual scheme which identifies a phenomenon, individuates different categories, and classifies the instances of that phenomenon into those categories.

All three stages – identification, individuation and classification – involve us in trying to produce something that is rich and useful for explanatory purposes. This requires different things in different contexts. What makes for a successful taxonomy will vary, depending upon the underlying purpose behind our constructing one in the first place.

This is worth observing, because in various respects our taxonomy of end of life interventions does not share the characteristics of the paradigm cases. Pointing out the contrasts will help clarify our methodology, as well as defusing some possible objections in advance.

In the paradigm case of speciation, we might think that the phenomenon is given: we are presented with the class of organisms, and aim to explain the development of and variation between those organisms. The process of individuation and classification is an attempt accurately to capture the real internal structure of that phenomenon: to ‘carve the world at its joints’, to borrow Plato’s phrase ^23^. A taxonomy with this representational ambition must capture pre-existing distinctions, and in so doing be both exhaustive and non-overlapping.

However, our aim is *constructive*, not *representational*. Instead of seeking to ‘carve at the joints’, we are creating a categorization for practitioners and theorists thinking about end of life issues (mainly, but not restricted to, end of life care). Therefore, the taxonomy we propose is broadly functional, and the standard it must meet is pragmatic. We ask: is it useful to think about things in these terms, to deploy these concepts, and to structure policy and practice around these categories? Given that the taxonomy does not have to be exhaustive, it can always be extended or amended at some future point if that would be useful. Nor is it problematic if its elements are overlapping, since many aspects of policy and practice have ambiguous or dual aspects. Indeed, we might think that the resulting ability to characterize ‘hybrid’ interventions is a strength of our proposed framework. Nor does it matter if some of our categories have an element of vagueness or indeterminacy; provided their inclusion helps others clarify thinking in ways that are useful. Ultimately, to borrow John Rawls’s evocative phrase, our aim is to secure an *overlapping consensus* ^24^ for end of life issues, identifying principles and concepts in which all citizens can share when thinking through the complex disagreements and challenges surrounding end of life services, practices, policies, values and beliefs.

Here we follow a trend in taxonomy creation within the social sciences ^25^, which has been seen as an exercise in bringing clarity and definition in emergent and complex circumstances, by forging a ‘common language … that distils complex interventions into their essential components’ ^26^. The most ambitious deployment of taxonomies in this context comes in moving from a passive description of elements, to a more active and critical appraisal of their veracity, in ways that enable us to plan for change ^27^.

In this spirit and in what follows, we therefore construct a taxonomy of *interventions* at the end of life, defined as ‘organized responses to end of life issues’. Our starting point is to observe the diversity of real-life interventions that take place, and then to build the taxonomy by ranging over what constitutes an ‘organized response’ and what can be categorized as an ‘end of life issue’. This work has been carried out inductively through wide reading, intense multi-disciplinary team discussion and debate, and through presentation to and feedback from expert international audiences in conference settings. It is not the product of a defined method (for example, the Delphi technique ^28^, with its emphasis on forecasting, was not thought suitable). Rather we have used inductive reasoning to generate categories from a wide range of extant data, covering many end of life issues, and recognising the uncertainties surrounding our conclusion. Some categories of intervention were obvious and well documented, others appear to be new and unfamiliar *as categories*. However, the resulting overall conceptual framework is new. It distinguishes different types of end of life intervention, providing in each case a free-standing working definition. It acknowledges that some interventions defy a single categorization, but (as noted above) we see this is a strength of our taxonomy, acknowledging explicitly the over-lap and ‘hybrid’ aspect of some specific interventions. We also seek ways to cluster the categories within the taxonomy where there appears to be a particular ‘elective affinity’ between them.

The resulting taxonomy focuses on end of life interventions in society, conceived broadly and globally. It takes in debates about end of life *care* but is not restricted to them. This is important: our ambition is to construct an overlapping consensus of principles, and the best way to do this for end of life care is to situate our thinking within a broader societal context. We discuss this synoptic ambition further in the conclusion, once our taxonomy is in place. **

## Findings

Using the approach described, our analysis generated 10 categories of end of life intervention. For each category we formulated two particular features: the ‘focus’ and the ‘locus’. The 10 categories of intervention were also clustered into two overall types – ‘frames’ and ‘instruments’.

### Categories of intervention

The 10 intervention categories captured a wide spectrum of actions and activities relating to end of life issues. End of life interventions can take many forms. Following a well established principle of social science analysis, our classification takes in macro, meso, micro and individual dimensions ^29^. It ranges from examples of interventions at the societal level, to those which exist in particular organisational or jurisdictional settings, to those which are highly localised in specific places, and those which are shaped primarily by persons, rather than larger groups or structures. Some interventions are well-structured, visible, highly documented, monitored and resourced. They exist in the public spaces of policy, service organizations and discourses, and are subject to their own forms of governance and accountability. At the opposite pole, are interventions that are loosely defined, uncodified, inchoate, partially visible or even hidden from view, existing in the more private or circumscribed spaces of localities, communities of interest, sub-cultures and social movements.

In Table 1, we offer formal descriptions of each category of intervention and provide some examples for each. In presenting the 10 categories, we give indicative references by way of illustration, but we acknowledge that subsequent work is required in order to examine the categories in more detail, moving from the ‘road map’ of the present paper to a more evaluative, situated and concrete position.

**Table 1.** Ten categories of end of life interventions.

	Focus	Definition	Examples
1	Policy	Decisions taken or rules adopted by governing authorities to deliver, facilitate, monitor or regulate end of life issues.	Strategies, regulatory and monitoring frameworks for end of life care, resource allocation protocols, standards, and guidelines.
2	Advocacy	Expressions or actions on end of life issues that aim to influence decisions of the institutional elite and/or promote the interests of specific populations, groups or individuals in particular contexts.	Calls for legalisation of medical aid in dying, assisted suicide, or euthanasia, concerns about inadequate access to pain medication or hospice and palliative care, ‘Declarations’ of various kinds on end of life issues.
3	Educational	Development of knowledge, skills, good judgment and character required for the delivery of appropriate end of life care	Educational resources and programmes extending from professional audiences to lay, family and informal carers and wider publics and interest groups.
4	Ethico-legal	Frameworks included within laws, guidelines or ethical codes that relate to issues at the end of life and which permit, facilitate or require specific courses of action.	Laws on with-holding or withdrawing treatment, assisted dying, euthanasia, suicide or the provision of pain relief and palliative care, professional requirements and standards about these issues.
5	Service	Medical, nursing and other services for the prevention, alleviation and/or reduction of suffering at the end of life through inpatient, outpatient, home care or other forms of services	Palliative care, hospice, pain and bereavement services, provision for housing with care, institutional and community based, public, private, non-profit.
6	Clinical	Medical, nursing, allied health and psycho-social procedures at the individual level to relieve symptoms and sufferings associated with advanced illnesses and when death is imminent	Procedures for pain relief, symptom management, care planning, bereavement care, for adults and children.
7	Research	Systematic enquiry on end of life issues for the purposes of establishing new knowledge and understanding by description, prediction, improvement and/or explanation	Studies in many disciplines and methodologies intended to illuminate, evaluate or re-define end of life issues.
8	Cultural	Initiatives taken to influence patterns of shared knowledge and symbolic meanings in particular communities, through which people perceive, interpret, express and respond to end of life issues	Designated ‘days’ relating to end of life issues, death cafes, salons, works of art, literature, film, poetry on end of life issues.
9	Intangible	Actions to promote the recognition and significance of aspects of human existence that have intrinsic value at the end of life	Spiritual care, therapies to promote dignity and compassion, to enhance the meaning of suffering, provide mutual support.
10	Self- determined	Actions, decisions or choices made by individuals to engage in or refrain from something that has implications for them at the end of their life or the life of another	Voluntary refusal of life prolonging procedures, treatment, food and fluids, ‘rational suicide’, self-care and support.

***1. Policy interventions.*** Decisions taken or rules adopted by governing authorities to deliver, facilitate, monitor or regulate end of life issues make up the category of policy interventions ^30^. They are among the most visible and structured forms of end of life intervention, with potentially a high impact, as they tend to relate to whole populations. Policy interventions about end of life issues include strategies, resource allocation protocols, standards, and guidelines that operate for specific groups of people ^31^. They cover areas such as the provision of palliative care across the age ranges, housing with care for older people, and support after bereavement. They can emanate from departments of government, as well as from professional bodies engaged with health and social care, and also from non-government organizations of various types ^32^. They can also include more conceptual interventions, such as the proposition that end of life care is a ‘public health issue’ ^33^ and that population-based planning should proceed on this basis.

Within the field of end of life care, and particularly when viewed from the position of palliative care protagonists, policies to support such provision are widely viewed as key determinants of recognition or viability for the field ^34^. The existence of national, federal or local policies for end of life care is commonly used as a marker of development, for example by the WHO in *Palliative Care for Noncommunicable Diseases: a global snapshot in 2015* (2016: http://www.who.int/ncds/management/palliative-care/palliative-care-NCDs/en/; accessed 25 January 2017). Conversely, in jurisdictions where such policies do not exist they are seen as key interventions necessary to advance provision.

Policies are revealed through texts, practices, symbols and discourses that define and deliver values, goods and services and regulations ^35^. There are examples of such materials from around the world as they relate to end of life care, but these are nowhere collated, analysed or critiqued. Crucially, there are very few instances of publically available monitoring and evaluation of end of life care policies at the national level ^36, 37^.

***2. Advocacy interventions.*** Expressions or actions on end of life issues and end of life care that aim to influence decisions of an institutional elite or promote the interests of specific populations, groups or individuals in particular contexts constitute advocacy interventions ^38^. These typically include agenda setting, lobbying, awareness-building, public mobilization, progress monitoring and in some instances case level action ^32^. End of life advocacy ^39^ can be found in relation to areas, for instance: calls for the legalisation of medical aid in dying, assisted suicide, or euthanasia; concerns about inadequate access to pain medication for those with advanced disease; the incomplete provision and coverage of hospice and palliative care; demands for greater awareness of bereavement at the policy level; as well as the work of organizations focussed on research and care relating to specific, sometimes rare, diseases. ‘Declarations’ of various kinds make up a very specific subset of advocacy interventions and seem to have proliferated in recent years, though little is known about their consequences ^40^. In the field of palliative care, the most significant of these, as noted earlier, is that of the World Health Assembly in 2014, calling on all its members states to integrate palliative care provision across the life course into their health policies and planning ^16^.

Advocacy groups supporting assisted dying or euthanasia have proliferated across many countries, though unlike the palliative care groups they are less likely to have international modes of organisation and activity. There appears to be little evidence about the direct effects of these groups upon changes in legislation. A specific element of advocacy interventions relates to the issues surrounding access to opioid medications and the ‘balance’ between global and national policies that regulate and restrict access in order to avoid misuse, in relation to those which facilitate access for medical purposes ^41^. There have also been alliances that bring together regulators with clinicians and civil society organisations campaigning for better opioid availability and access ^42^.

***3. Educational interventions.*** Development of knowledge, skills, good judgment, and the character required for the delivery of appropriate end of life care and other end of life activities are key aspects of educational interventions ^43^. This potentially very large category of educational interventions involves the development of resources, delivering programmes and maintaining their quality, accreditation, and improvement. It comprises many pedagogic constituencies and methods. It is delivered by many types of organization, formally as well as informally. It has the potential to reach large audiences through open learning and web-based technologies. It extends from professional audiences to lay, family and informal carers and also reaches out ultimately to schools, wider publics and interest groups.

The promotion of educational interventions on end of life issues is often an aspect of policy and advocacy work, demonstrating again the cross-cutting nature of some of the categories in the taxonomy. Specific educational programmes often include evaluation and feedback methodologies and there is some knowledge about the scope and reach of these, particularly when they operate ‘at scale’ ^44^. Overall however, despite the massive investment of resources in education and training on end of life issues, there is little sense of its macro purposes and impact. As ‘open’ and ‘lifelong’ learning become increasingly valued, there is scope for the critical review of why, how, for whom and with what benefits such interventions take place. It is neither perverse nor fanciful to pose the question: why end of life education?

***4. Ethico-legal interventions.*** Frameworks within laws, guidelines or ethical codes that relate to issues at the end of life, and which permit, facilitate or require specific courses of action make up a separate category of interventions. These include laws on the with-holding or withdrawing of treatment, assisted dying, euthanasia, suicide or the provision of pain relief and palliative care – which may in turn relate to policy or advocacy on the same issues. They also include professional requirements and standards relating to the use of pain relieving or other medications at the end of life, for example in relation to the practice of sedation ^45^. In such areas, a close overlap is found with specific end of life clinical interventions. Globally, we lack an overview of such interventions. The World Medical Association has made a series of statements on ethico-legal aspects of end of life care, but at a national level it is less clear how medicine and the health professions are responding, and whether or not there is broad parity in the positions taken, or significant differences. The most marked source of variation is in relation to the small number of countries or states that have intervened to legalise some form of assisted dying, and those that have not.

***5. Service interventions.*** From the last quarter of the twentieth century there has been a significant rise in the numbers of medical, nursing and other services for the prevention, alleviation and/or reduction of suffering at the end of life through hospital inpatient and outpatient facilities, care in the community, in hospices and nursing homes or other forms of provision. Within the literature, the growth and distribution of such services globally, as well as within specific regions and countries, has attracted some attention. There is a body of knowledge for each country in the world about the development of services specifically for the delivery of hospice and palliative care ^9, 46^, though the methods of mapping and ranking require refinement and review ^47, 48^. The development and delivery of end of life care services is closely linked to local funding and reimbursement arrangements, and is sometimes the territory of a mixed economy of publically funded, for-profit and non-profit organisations. There is also a significant interest, but no consensus, in models of service organisation and delivery, with particular enthusiasm for identifying ‘scalable’ models, which can meet changing population need and are sustainable over time ^49^.

***6. Clinical interventions.*** Medical, nursing, allied health and psycho-social procedures at the individual level to prevent and relieve symptoms and sufferings associated with advanced illnesses and when death is imminent, as well as following loss, make up an array of clinical end of life care interventions that first began to be codified in the nineteenth century ^50^. These include interventions for pain, such as the WHO ‘pain ladder’ for adults ( *WHO,* 2013: http://www.who.int/cancer/palliative/painladder/en/; accessed 25 January 2017). They also include interventions for symptom management, plus techniques to promote quality of life and individual strategies to improve communication, co-ordination and care planning. They sit in the specialist territory variously described as ‘palliative’, ‘hospice’, ‘end of life’ and ‘bereavement’ care; but increasingly they overlap with other medical specialties, not only oncology, but also geriatrics, cardiology, neurology, paediatrics, orthopaedics and psychiatry. Perhaps more than their service-level counterparts, clinical interventions have generated a body of research evidence, as well as extensive commentary, guidelines and standards for optimal care. Nevertheless, there are ongoing concerns about the strength of the evidence base for clinical interventions at the end of life – and a growing interest in the distinction between ‘specialist’, ‘generalist’, and ‘early’ involvement in these areas. We also recognise that some clinical interventions at the end of life have attracted negative scrutiny and evaluation. The development, policy endorsement, roll out and subsequent withdrawal of the Liverpool Care Pathway in the United Kingdom is a case in point, and demonstrates the difficulties that can occur when interventions well understood and demonstrated in one context (hospice) can run into problems when applied at a system level in another (acute hospital) ^51^.

***7. Research interventions.*** Systematic enquiry into end of life issues for the purposes of establishing new knowledge and understanding, by description, prediction, improvement and/or explanation, can be regarded as a distinct form of intervention. End of life research interventions include setting research strategies, the provision of dedicated research funding, undertaking empirical or theoretical studies, and the dissemination of findings through knowledge exchange and implementation strategies. Research is included here as a category of intervention because it must be regarded as *constitutive*, as well as analytical and descriptive in character. In other words, research on end of life issues not only uncovers ‘facts’ or ‘evidence’ about those issues, it simultaneously shapes and frames them in particular ways. Some of this can be seen in the methodological and practical debates that go on within end of life research ^52^, but this is more than an argument about the power of the methods or the hierarchy of resulting evidence. It extends to definitions of the situation that determine what can and should be studied, how this relates to unfolding policy and practice, and the power and political dynamics of knowledge production. End of life research interventions themselves require analysis – to tease out what is prioritised, what claims to knowledge are made, as well as silences and absences in the chosen areas of enquiry.

***8. Cultural interventions.*** Initiatives taken to influence patterns of shared knowledge and symbolic meanings in particular communities, through which people perceive, interpret, express and respond to end of life issues, make up a wide array of cultural interventions. In this part of the taxonomy can be found activities that are not only recent in origin, but which may in other paradigms not be considered to constitute ‘interventions’ at all. Included here are social and artistic events and activities, public engagement on end of life issues, exhibitions, film, theatre, and the use of social and mass media, death cafes ^53^/salons, and named or themed days/weeks focussed on matters of dying, death and bereavement.

In considering these interventions, we are thinking beyond the broad array of cultural practices, traditions and rituals associated with the end of life that exist in all cultures and societies to some degree, although a case may well be made that these too are ‘organized responses’. Rather we seek to focus on the structured use of art, culture, and imagination to raise questions, challenge existing viewpoints, provide opportunities for reflection, promote debate, and foster engagement with questions of mortality, care at the end of life, and associated values, preferences and meanings.

This appears to be a burgeoning set of end of life interventions, developing rapidly in the early years of the 21 ^st^ century, but building on many preceding cultural forms. Unlike policies, services and clinical interventions, its rules of engagement are not tied up with standards, outcomes and measurability. Its protagonists might argue that these forms of intervention seek to do something more challenging than most others – to influence cultural practices, beliefs, expectations and values in society. Cultural interventions also have the wherewithal to engage with complex ethical, moral and political issues, such as assisting someone to die, dealing with loss after terrorist attack, exploring the end of life experiences of migrants, addressing the end of life concerns of lesbian, gay, bi-sexual and transgender people, or reactions to the death of an infant, neo-nate or unborn child.

We contend that these interventions are largely ignored by the gaze of policy makers, researchers, pedagogues, service deliverers and clinicians. Yet when cultural interventions are linked to any of these topics, the effects are also important. For those involved, they may be largely of expressive, rather than instrumental, value. At the least, cultural interventions on end of life issues seem to merit more attention, elucidation and analysis. They are currently underserved by the interests of researchers and cultural critics.

***9. ‘Intangible’ interventions.*** Actions implemented to promote the recognition and significance of abstract and non-physical aspects of human existence, which have intrinsic value in end of life issues, make up the category of intangible interventions. This category is inspired by work undertaken by the ATLANTES Programme, at the University of Navarra: *Intangible Aspects of Palliative Care* ( https://www.unav.edu/en/web/instituto-cultura-y-sociedad/proyecto-atlantes/investigacion/intangibles; accessed 25 January 2017). It includes spiritual care interventions, as well as opportunities to explore meanings, beliefs and values on the part of those giving and receiving care. It can also include attitude formation, dignity preservation and the fostering of compassion around end of life issues. Some of these interventions might equally be included in the ‘services’ and ‘clinical’ ^54^ categories described here. Some cultural interventions may also touch on ‘intangible’ aspects.

In particular, this category challenges our capacity for understanding and interpretation, and calls for approaches that are sensitive to the context and fine-grained character of such interventions. Whilst often seen as an under-resourced and poorly acknowledged area, there are some examples of groups, initiatives ^55^ and taskforces ^56^ that promote the understanding and practice of intangible interventions.

***10. Self-determined interventions.*** This category is made up of actions, decisions or choices made by individuals to engage in or refrain from something that has implications for them at the end of their life or the life of another. Self-determined interventions include refusal of life-prolonging procedures, declining treatment, the voluntary refusal of food and fluids, ‘rational suicide’ and the use of self-chosen technologies to bring about one’s own death. They also include a wide array of self-care and support and actions directed towards making one's life with illness more comfortable or more expressive of one’s identity. They can also include advance care planning and making plans for death.

This category of interventions has some particular characteristics. It comprises actions taken by individuals of their own volition, rather than being subject to actions determined by others. It is a window into human agency on end of life issues, where the actors are not in any professional role. Self-directed interventions are also an ‘organized response’ in the sense that they make up a definable class of actions that are governed by ethical and moral principles of choice, rationality and freedom, or alternatively, constraint, duty and obligation. In some cases, though not all, interventions of this type are shaped too by the discourses and strategies of advocacy. They are also likely to be directly influenced by cultural interventions and considerations. Whilst professional practices, policies and guidelines, research and education combine to transmit what is known about more formally constituted service and clinical interventions, remarkably little attention has been given to those which are in large degree ‘self-determined’.

Box 1. **Policy points**

- End of life issues are beginning to figure more strongly in public debate and planning, but we lack understanding of new approaches to these matters, beyond those that sit within the paradigm of hospice, palliative care and related health and social services. There are many other forms of intervention at the end of life that merit policy, practice and research attention.
- Our goal is to generate a taxonomy of end of life interventions that has four distinct benefits: 1) over-viewing the many forms of organized human endeavour that are oriented to end of life issues; 2) mapping the terrain into relatively distinct elements; 3) alerting sectional interests and specific stakeholders involved in end of life interventions to the range of cognate actions that exist and providing opportunities for synergy, partnership or complementarity; 4) providing a framework for the macro-evaluation and synthesis of knowledge across the spectrum of interventions.
- To date the ‘end of life’ field has prioritised empirical ‘evidence’ and ‘common sense’ argument over conceptual reasoning and critical analysis. The taxonomy of end of life interventions presented here could be the first step in a paradigm shift towards a more rigorous and comprehensive understanding, fostering mutual learning and better policy making.

## Discussion

The character and location of any intervention are each important dimensions for its understanding. We characterise these as the ‘focus’ and the ‘locus’. In addition, the predominant orientation of the intervention needs to be considered – whether it is to illumination and ‘framing’ or to direct engagement as an ‘instrument’ for action. We consider each of these elements.

### Focus and locus – practical dimensions

Focus refers to the character of the intervention. It concerns the elements found within it, the field of objects to which it is addressed and the related purpose of intervening. Focus is about the content, orientation, and qualities of the intervention. It can also include the goals and ambitions of those who construct and deploy the intervention. The focus of any intervention may change over time or as it shifts from one locus to another. Within our schema, focus means more than objectives or goals, or indeed what is known about the success of an intervention in achieving these. We contend that understanding an end of life intervention must go beyond this and can profitably include some account of the motivations of its instigators, the processes of its implementation, the field of discourse in which it is located and the presence or absence of unintended consequences relating to it.

Locus refers to the spatial dimension of the intervention. Some interventions are developed and implemented on a global scale. Others are oriented to a specific set of countries. Some are designed for a particular jurisdiction or a region or locality within it. Any given intervention may have the potential to move across different *loci* and this also creates important questions about the consequences that result. This is particularly relevant in contexts where policy transfer or policy mobility is part of the intended aim of an intervention. Likewise, interventions which ‘travel’ and those which remain in one location, are also worthy of comparative consideration. Whilst there is considerable enthusiasm for the ‘roll out’ of ‘scalable’ interventions of proven efficacy, there is much less attention to the modalities of transfer and the potential for interventions to be translated into local contexts in ways that might then feedback to and transform the original conceptualisation. More attention should be given to the indiscriminate transfer of specific end of life interventions from one locus to another, without due diligence, appropriate foresight and a sense of ‘responsible innovation’.

### Frames and instruments – conceptual aspects

**Figure 1.**
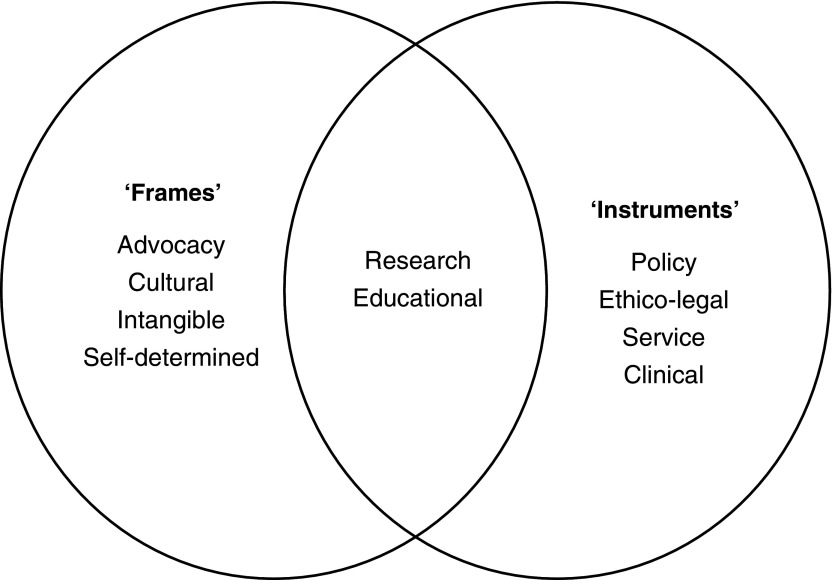
End of life interventions as ‘frames’ and ‘instruments’.

How to make conceptual sense of this array of intervention types? We find it helpful to distinguish between interventions as ‘frames’ and as ‘instruments’. ‘Frames’ primarily *construct* a shared understanding of an end of life issue. They are about illumination, suggestion, and characterisation of particular themes and allow for high levels of difference and disagreement as well as ‘emergent’ phenomena in changing social contexts, accordingly their boundaries might be blurred. ‘Instruments’ are organized responses which *assume* a shared understanding and then move to act in that context. Whilst they remain subject to verification by scientific methods, they nevertheless operate within more fixed paradigms of knowledge and evidence and to this extent they have clearer boundaries. This distinction between ‘frames’ and ‘instruments’ is for heuristic purposes only. It is not intended to define two separate categories of intervention. Rather it alerts us to separate properties that can be more or less present in any one category. Figure 1 shows how the 10 categories can be broadly distinguished from this perspective, but these are schematic, not absolute, distinctions.

## Conclusions

We take the view that end of life interventions, despite their range and variation, can be categorised in ways that are useful. We offer a10 point taxonomy of end of life intervention, along with two sets of cross-cutting themes: ‘locus’ and ‘focus’, plus ‘frames’ and ‘instruments’.

We argue that ‘organized responses’ to end of life issues is a workable definition for end of life interventions. In doing this we recognize that the level and character of the ‘organization’ in question differs significantly. The types of organization involved in intangible or self- directed interventions are not those found in service, policy or clinical interventions. We acknowledge that some interventions are organized through the mechanisms of culture and beliefs, others through legal or professional frameworks, or formal organizational structures. We can see considerable scope for further reflection and conceptualisation on some of our categories, case by case. For example, ‘intangible’, self-determined’, and ‘cultural’ interventions are emergent in end of life discourse and therefore need further exploration. Whereas ‘advocacy’ and ‘policy’ interventions relating to the end of life are fairly well established, but lack an evidence base.

There is a broad consensus that in general, interventions relating to health and social care should be evidence based and subjected to correct implementation processes that can be measured. This ‘gold standard’ is a far cry from most of the categories of intervention we have identified. First, it is difficult to conceptualise how evidence and monitoring would be applied to some categories of intervention in the taxonomy. Indeed, such an approach might be inimical to the spirit and rationale of the intervention itself. In some instances, the intervention might be of doubtful legal status, leading to secrecy or reluctance to ‘allow in’ the mechanisms of surveillance required for evaluation and monitoring. In certain jurisdictions, self-determined interventions to voluntarily withdraw from the intake of food and fluids or to actively assist in the death of another at that person’s request would be examples of this type. Second, it is clear that even when policies, services and clinical interventions sit within a paradigm where evidence-based approaches are to be expected, major challenges also exist. As one commentator recently remarked ‘no current policy or practice designed to improve care for millions of dying Americans is backed by a fraction of the evidence that the Food and Drug Administration would require to approve even a relatively innocuous drug’ ^57^. So, we recognise that many end of life interventions within health and social care lack an evidence base for efficacy, safety or implementation, and we argue that other factors must therefore be taken into account when making sense of why, how and with what impact they are introduced.

In this context, we have provided a *conceptual platform* from which to gain a wide overview of the broad and developing field associated with end of life interventions. It is clear that this field has many dimensions. Overarching them all is a sense of concern and engagement with end of life issues, often in cases where there is conflict or contestation around appropriate courses of action. Much of that is about approaches to end of life ‘care’, but some of it is also about the meaning of death in contemporary life, along with associated attitudes, beliefs and values. We have explained that many interventions at the end of life relate to the discourse of global health, which is committed to the reduction of inequities and to the value of mutual learning. By studying interventions from the viewpoint suggested here, it may be possible to better understand the significant ‘gaps’ in provision that exist, but also more effectively to reduce them.

In mapping this terrain, we have chosen to narrow our enquiry around the concept of ‘intervention’. This involves some stretching of the concept beyond its usual meaning in the policy literature, but we see value in this – showing how interventions can have many starting points and may vary in their goals, ambitions and mechanisms. We think the approach highlights the importance of alerting sectional interests to cognate endeavours in the end of life field. Our approach gives value to interventions that might otherwise be marginalized or over-looked. In turn it challenges the biomedical power structures of ideas, practices and resources that so often seek to dominate the discourse around end of life issues.

At the same time, we provide a framework for thinking about macro-evaluation and synthesis. This requires us to go beyond current methodologies and hierarchies of evidence. At this point in the evolution of the end of life ‘field’, we take the view that more critical reflection, under-pinned by appropriate theories and modes of reasoning, can make an important contribution. Put candidly, the field is under-theorised and to date has prioritised empirical ‘evidence’ and ‘common sense’ argument over conceptual reasoning and critical analysis. The taxonomy of end of life interventions presented here could be the first step in a paradigm shift towards a more rigorous and comprehensive understanding.

This theoretical ambition is matched by the aspiration to unite academic theory with broader political and social practice. We noted that our ultimate aim is to help secure an ‘overlapping consensus’ for end of life issues, echoing John Rawls’s influential phrase. We conclude by exploring this more fully, as a way of showing how the taxonomy described here can have important practical consequences.

Rawls used the notion of an overlapping consensus to identify how we might construct legitimate rules for the exercise of power in the face of disagreement over fundamental matters of justice, religion, morality and culture ^24^. In multicultural and increasingly globalized societies, those disagreements are deep and persistent. Moreover, they are reasonable. They are not the result of individuals or groups being immoral or obtuse, but instead reflect how they come to these questions with different values and identities, and that honestly grappling with such complexities can lead to differing answers. So, this disagreement commands a kind of respect. In particular, societal rules governing the exercise of power are legitimate only when they are such that ‘all citizens … may reasonably be expected to endorse in light of the principles and ideals acceptable to their common human reason’, in spite of all the other deep respects in which they disagree ^24^. To do this, Rawls says we must limit ourselves to principles that are either shared by all reasonable citizens, even if they disagree about other things; or to principles that can be derived and grounded in that shared overlapping territory. It is not always easy to identify that shared ground, and we might worry that it gets determinate content only by smuggling in deep (and potentially controversial) commitments that run counter to the value-neutrality that Rawls ostensibly endorses ^58, 59^. But, if we can face up to this task, then we will get a set of principles which allow us simultaneously to respect deep and sincere disagreements, while also bracketing them so that we can build practical convergence on rules to govern our society.

Rawls’s theory deals with principles of justice: that is, societal rules of the most general form. But, we can deploy the notion of an overlapping consensus in more restricted domains as well. Take any area of public policy wherein we (as a society) must converge on rules about what to do, but which is also characterised by deep and persistent disagreement based in moral, religious, philosophical and cultural convictions. In this context, the best way to proceed is to focus on principles that we can share, which are part of the overlapping consensus between (otherwise divergent) convictions, or can be brought into that consensus through shared public reasoning.

An illuminating (and heartening) comparison here might be drawn with the Capabilities Approach developed by Amartya Sen and Martha Nussbaum, as a framework for thinking about human development goals ^60^. An individual’s capabilities represent his or her effective freedom to do or be various valuable things. Sen and Nussbaum identify a number of valuable functionings, which are multiply realisable: they can be expressed in different ways by different individuals. For example, Nussbaum insists that bodily health, social interaction and political engagement are all important capabilities, which we must seek to guarantee for all individuals; but we must do so in ways that recognise that, for example, those social interactions that are valuable and appropriate will vary between contexts ^61^.

This inherent flexibility allows us to converge on practical measures needed to support capabilities, while sidestepping cultural and individual differences concerning the specific ways in which those capabilities might be used. The Capabilities Approach has been strikingly successful in generating consensus around how to tackle global health inequality, creating a set of shared principles and international development priorities, despite continuing deep disagreements on cultural, religious, moral and political norms ^62^.

The notion of an overlapping consensus can play a similarly vital role in assisting convergence on a framework for dealing with end of life issues. Rawls’s notion focuses on the value-commitments that might be shared. But it is equally important, especially in a fraught and obscure topic, to develop a shared conceptual apparatus that can frame and illuminate public reasoning about what to do.

Our taxonomy makes a contribution to identifying and expanding an overlapping consensus that can generate practical convergence, which respects this disagreement without being paralysed by it. It gives us a flexible structure that allows us to concentrate on areas of intervention where there might be local convergence (e.g. on service and clinical activities in geriatric and palliative care), even when there is further discussion needed on other areas (e.g. the wider legal framework, or on cultural attitudes to death and dying). In addition, it contains concepts – especially those of ‘intangible’ and ‘self-directed’ interventions – that are fertile and open-ended, and thereby give practitioners and other researchers space to develop ideas that can build agreement in specific cultural and philosophical contexts. There is, no doubt, further work to be done, both in elucidating our thinking and in developing our practice in the end of life domain. A constructive taxonomy of end of life interventions is an essential starting point for that further work. And this is what – through a rough-and-ready mixture of empirical description, conceptual analysis, and functional reflection – the present paper aims to provide.
