# Supplementary material for: Interventions at the end of life – a taxonomy for ‘overlapping consensus’
Source: Wellcome Open Res. 2017 Feb 2;2:7. [Version 1] doi: 10.12688/wellcomeopenres.10722.1 (PMC5336190; doi:10.12688/wellcomeopenres.10722.1)
Supplement: Peer review history

# Peer review: Referee response for version 1

**DOI:** 10.21956/wellcomeopenres.11562.r19889

**Published:** 2017-02-28

**Authors:** Mary Ellen Macdonald, Renata Bubadué, Raissa Passos dos Santos, Martine Lévesque, Mark Keboa, Harprit K. Singh, Basem Danish, Gail Teachman, Crystal Noronha

**Affiliations:** 1 Division of Oral Health and Society, Faculty of Dentistry, McGill University, Montreal, QC, Canada; 2 Pediatric Palliative Care Research, Montreal Children's Hospital of the McGill University Health Centre, Montreal, QC, Canada; 3 Views on Interdisciplinary Childhood Ethics, School of Nursing, McGill University, Montreal, QC, Canada; 4 Faculty of Dentistry, McGill University, Montreal, QC, Canada; 5 Department of Philosophy & Biomedical Ethics Unit, McGill University, Montreal, QC, Canada; 6 Views on Interdisciplinary Childhood Ethics, Centre for Research on Children and Families, McGill University, Montreal, QC, Canada

**Strengths: **We are very excited and impressed by this article. The work of the team is extensive; the topic important; the findings impressive and scholarly. The Background section is particularly well documented and encompassing, nicely setting up the context for this work. We appreciated the combination of pediatric and adult palliative care concerns. We were especially moved by the uncovering of global inequities and injustices (e.g., pain medication), and impressed with the diversity, specificity as well as critical engagement enabled by the taxonomy (and we applaud the resistance to simplification!). We were also appreciative of the careful attention to scholars such as Rawls to help conceptualize the project.

**Suggested revisions: **Given that we feel this paper has the potential to make a substantial contribution to palliative care research, we spent considerable time grappling with how best to convey its strengths. Ultimately, we feel more work is needed to convey clearly the creative yet rigorous process involved in creating the taxonomy, while also underscoring the scholarly potential of this work. It feels to us that there may be two papers contained in this one manuscript. While we leave this to the authors to decide, we do feel that dividing the current work into a) a more detailed methodological section, followed by b) a section that then discusses the true potential of this provisional taxonomy (e.g., as a tool for synergy, synthesis, elaboration, and evaluation) may be more fruitful. Details follow.

  

- **The appropriateness of the title**

**Title: **As suggested above, we believe there are multiple elements contained in this one manuscript. The title is descriptive of only one, however: the presentation of the taxonomy. It thus misses the other elements (e.g., the rigorous development of a creative new tool; the potential utility of this tool for the field of palliative care globally).

  

- **Whether the abstract provides an adequate summary of the article**

**Abstract: **Our concerns with the Abstract parallel our concerns with the paper (as outlined more fully below). Currently, the Methods section defines the term “interventions” yet lacks description of the methods. The taxonomy is described as “comprehensive” which seems inconsistent with the more provisional language used in the text (e.g., p5, para1: the authors note the taxonomy is not necessarily exhaustive and is open to future elaboration). The Abstract locates methodological decisions (e.g., conceptualizing categories with the heuristics: locus, focus, frames and instruments) in the Findings and thereby downplays their roles in actually shaping the Findings. Similarly, topics we feel should be developed in the Discussion section (e.g., Rawls on overlapping consensus) appear in the Conclusion. [And note: the use of the word ‘range’ to describe the interventions is perhaps incommensurate with the conception of overlapping consensus.]

  

- **If there is a comprehensive explanation of study design, methods and analysis, and their suitability to the investigation**

**Methods: **We would like to see a more robust discussion of the creative process used to develop this taxonomy. The iterative methodology employed has clearly produced a thoughtful tool with great utility. We feel that it behooves the authors to more clearly distinguish the data gathering from the conceptual processes used in its development. The main Objective is “to generate a taxonomy of end of life interventions wherein they are ordered and named in a system of classification” (p4; para5). While this taxonomy has been generated, the actual processes of that generation are less clear. The metaphor of the “road map” is used (p5, para7); however, we feel more careful explanation of this mapping is required. This descriptive work will better set up the authors’ future goal of establishing “a more evaluative, situated and concrete position” (same paragraph).

 For instance, a Table itemizing the data sources would have great utility. Currently, claims of ‘global’ coverage and ‘wide reading’ are not well evidenced given the reader does not have access to the data sources. Reference is made to the kinds of data sources (p5, para3); more detail is needed, at least in broad strokes (perhaps in an Appendix).

 We suggest then moving the “four distinct benefits” (p4, para5) to the **Discussion**, and using these as a scaffold for the **Discussion**. Currently, what appears in the **Discussion** is actually evidence to back up the analytic decisions made and thus would have more utility in the **Methods** section. This information will help explain how the empirical data was parsed from the conceptual processes in the category development.

 [And note: we believe that what is currently in the **Conclusion** (e.g., the concern about the evidence base, the critique of under-theorized, biomedically-focused end-of-life research, and the utility of Rawls’ work) is actually important fodder for the **Discussion**.]

 Similarly, reference is made in the **Findings** to key information that assisted the methodological decisions. For example, p5, para5 makes reference to focus vs. locus; para6 refers to the macro, meso, micro and individual dimensions of the classification; and later in the **Discussion** is reference to frames vs. instruments. Referencing and discussing these important constructs (called ‘themes’ in the **Conclusion**, para1) outside the methods section downplays their roles in shaping the 10 categories; this placement thereby also obscures how inductive or deductive the category development was.

 An aside for consideration: Distinguishing the taxonomy-building methodology from a more conventional methodology (e.g., a scoping review) could also be helpful. This comment is both to underscore the originality of the team’s choice, while also to help orient the reader to the **Findings**. Currently, there are references throughout the **Findings** which signal empirical data (e.g., dignity therapy) as well as conceptual frames (e.g., philosophy of education). Following a scoping review methodology, one would expect only empirical data to be referenced in the **Findings**, perhaps with a Table at the end listing the data sources used. As currently presented, it is not always clear if the references indicate an intervention being categorized, or a conceptual justification for that categorization.

 Note also: The justification for the label “taxonomy” seems cumbersome and the foray into biological taxonomy and Plato is distracting. Perhaps simply finding reference to a kindred use of ‘taxonomy’ (e.g., from Nursing literature) could provide a clearer, more robust explanation and justification for the ability of this term to beget the constructive intention of the methodology, still with the Rawlsian intent. Further, an earlier explanation of how the notion of “overlapping consensus” was applied to or inspired the development of the taxonomy would enhance the reader’s comprehension. Though the Rawlsian concept is well explained in the **Conclusion**, understanding earlier how it applied in this work (e.g., for deriving the taxonomy or as a conceptual foundation for the whole project) would greatly contribute clarity.

**Findings: **Table 1 nicely covers the categories. We were uneasy, however, with the labels for Category 4, 9 and 10. #4: We questioned the neologism, “ethico-legal,” and would argue that more is lost than gained by lumping together these two domains in one word. #9: “Intangible” sets up a false dichotomy and can be seen to devalue these sorts of interventions. #10: The title “self-determined” may be too Western to have global reach.

  

- **Whether the conclusions are balanced and justified on the basis of the results**

See above.

  

- **With regards to the data (if applicable), whether sufficient information has been provided for replication of the experiment, and/or if the data are in a usable format**

See above.

  

- **Miscellanea**

For references from websites: we suggest moving the website details and ‘dates accessed’ to the reference list.

We have read this submission. We believe that we have an appropriate level of expertise to confirm that it is of an acceptable scientific standard, however we have significant reservations, as outlined above.

# Peer review: Referee response for version 1

**DOI:** 10.21956/wellcomeopenres.11562.r19891

**Published:** 2017-02-23

**Authors:** Harry Yi-Jui Wu

**Affiliations:** 1 Medical Ethics and Humanities Unit, Li Ka Shing Faculty of Medicine, The University of Hong Kong (HKU), Pok Fu Lam, Hong Kong

This is an innovative paper written with clear structure, objectives and argument.

 It does offers researchers in the field a conceptual framework to consult or refer to.

 I believe that it is a foundational survey work that will lead to many other important projects. The main contribution of this paper is that it does identify the niche in the field that a grounded and evidence-based work is badly needed while policies, clinical guidelines, pedagogical plans and research agenda are being drafted or established. I would like to congratulate the authors of this paper for the groundwork they have laid down and the gateways they have paved.

 There are, however, several points I think authors might consider to improve the article. First is the methodology: the scope of the survey is extensive and ambitious. The authors also use several paragraphs signposting and explaining why the survey is important. However, in addition to “why” and “what” the categories of end-of-life issues are, it might be worthwhile to elaborate more regarding to “who”, “when”, and “how” the study in the end came across such taxonomy. Although the authors have described that the data are from the “wide reading, intense multidisciplinary team discussion and debate, and through presentation to and feedback from expert international audiences in conference settings”, and the selection stages of “identification, individuation and classification”, it will be more convincing if the authors could specify, e.g. How long was the study period? The examples of interventions collected spanned from when to when? In what countries were the examples collected? What databases are used? What are the analytical methods?

 In addition, it would be great if authors can detail how categories of interventions are singled out. Are there any coding methods employed? What are the criteria? Why ethico-legal interventions and clinical interventions must be distinguished categories… Aren’t they somehow the results of policy-making and advocacy?

 Second, in the introduction, the authors claim that the work aims to secure an *overlapping consensus *in John Rawls’ term. Is it the top instructive principle of data collection or the finding after data are collected? Readers might want to know if there are any findings that do not support the “overlapping consensus” discourse. In addition to the observed consensus, are there any contesting categories?

 There are also several minor technical problems that, I think, can be improved easily. In the article, the tones and writing styles of different parts are not consistent. The context part especially reads more colloquial. Apart from that, readers might be confused about what arguments are made in existing research, and what are suggested by authors in this paper. It also contains a number of redundant expressions. For example, (in the first page first paragraph line 3) “indeed”, (second paragraph line 1) “should”, (line 8) “as much as”, (paragraph 4 first line) “something that must”. It would be great to streamline these paragraphs. But these problems should be easily solved.

I have read this submission. I believe that I have an appropriate level of expertise to confirm that it is of an acceptable scientific standard.

# Peer review: Referee response for version 1

**DOI:** 10.21956/wellcomeopenres.11562.r20137

**Published:** 2017-02-22

**Authors:** Michael Connolly

**Affiliations:** 1 School of Nursing, Midwifery and Health Systems, University College Dublin, Dublin, Ireland

Thank you for the opportunity to read and comment on this interesting work. The title is clear and unambiguous and articulates what the article entails. The abstract provides and appropriate summary of the article giving a real sense of what to expect. The context is well constructed and sets out the space where the debate is taking place about palliative and end of life and death. The objectives are coherent and realistic in their intent. The method used to arrive at the taxonomy described is articulated well and with authority. The authors are intent on ensuring that what is presented is a guide based on evidence and expert discussion and opinion and should be taken as functional and pragmatic.

 I believe the taxonomy presented is a very balanced one which clearly focuses on what the authors describe as frames and instruments as a means of arriving at consensus to support the practical. The work in my opinion is timely and will be of interest to all those engaged in the work of palliative and end of life care and particularly those who are engaged in the discussions of how best we provide end of life interventions in the world today.

I have read this submission. I believe that I have an appropriate level of expertise to confirm that it is of an acceptable scientific standard.
